# The Role of Mannose-Binding Lectin and Inflammatory Markers in Establishing the Course and Prognosis of Community-Acquired Pneumonia in Children

**DOI:** 10.3390/children10111744

**Published:** 2023-10-27

**Authors:** Roxana Taraș, Beatrice Mahler, Mihaela Bălgrădean, Diana Derewicz, Miruna Ioana Lazăr, Ruxandra Vidlescu, Florian Berghea

**Affiliations:** 1 Department of Paediatrics, “Dr. Carol Davila” University of Medicine and Pharmacy, 050474 Bucharest, Romania; roxana.taras@umfcd.ro (R.T.); mihaela.balgradean@umfcd.ro (M.B.); diana.costache@drd.umfcd.ro (D.D.); 2 Emergency Clinical Hospital for Children “Maria S. Curie”, 077120 Bucharest, Romania; miruna-ioana.lazar@rez.umfcd.ro; 3 Department of Pneumophthisiology II, University of Medicine and Pharmacy “Carol Davila” Bucharest, 020021 Bucharest, Romania; beatrice.mahler@umfcd.ro; 4 “Marius Nasta” Institute of Pneumophthisiology, 050159 Bucharest, Romania; 5 Department of Internal Medicine and Rheumatology, “Dr. Carol Davila” University of Medicine and Pharmacy, 050474 Bucharest, Romania; florian.berghea@umfcd.ro; 6 “Sfânta Maria” Clinical Hospital, 011172 Bucharest, Romania

**Keywords:** mannose-binding lectin, community-acquired pneumonia, procalcitonin, C-reactive protein

## Abstract

Background: Community-acquired pneumonia (CAP) is one of the most significant childhood diseases worldwide and a leading infectious cause of death in children. This study aimed to evaluate the prognostic value of the inflammatory markers—C-reactive protein (CRP) and procalcitonin (PCT)—and the polymorphic glycoprotein mannose-binding lectin (MBL), deficiency of which is associated with severe infections, in the determination of the optimal type and timing of therapeutic intervention for CAP in childhood. Methods: Retrospective evaluation was conducted on a cohort of 204 children aged 4 months–17 years hospitalized with CAP. Their levels of CRP, PCT, and MBL were assessed for their association with a variety of outcomes, including the incidence of local and systemic complications, admission to the ICU, duration of antibiotic treatment and hospital stay, and death. Results: CRP and PCT proved to be better predictors of complications of CAP than MBL. The area under the curve (AUC) value was highest for PCT as a predictor of systemic complications (AUC = 0.931, 95%CI 0.895–0.967), while CRP (AUC = 0.674, 95%CI 0.586–0.761) performed best as a predictor of local complications (AUC = 0.674, 95%CI 0.586–0.761). Regarding admission to the ICU, CRP was the weakest predictor (AUC = 0.741), while PCT performed the best (AUC = 0.833), followed by MBL (AUC = 0.797). Sensitivity and specificity were calculated for the optimal threshold generated by receiver operating characteristic (ROC) curves, rendering sensitivity of 90% and specificity of 87% for PCT in assessing the risk of systemic complications, compared to sensitivity of 83% and specificity of 90% for CRP. MBL showed relatively high sensitivity (96%) but low specificity (25%) for predicting the need for ICU admission. Conclusions: Early measurement of CRP, PCT, and MBL provides clinicians with important information regarding the course and prognosis of children diagnosed with CAP, thus ensuring prompt, optimal therapeutic management.

## 1. Introduction

Pneumonia is one of the most significant childhood diseases and is the leading cause of mortality in children under 5 years of age, particularly in developing countries [1]. Mild forms of community-acquired pneumonia (CAP) usually respond to outpatient treatment, with complete recovery of normal lung architecture and function. Severe or recurrent forms of pneumonia carry the risk of developing long-term complications, such as postsurgical pleuropulmonary sequelae or childhood interstitial lung disease (chILD) [2]. Hospitalization for pneumococcal pneumonia is associated with a significant risk of complications, which occur in approximately 40% to 70% of cases, the most frequent being pleurisy, empyema, necrotizing pneumonia, lung abscess, and sepsis [3]. In the case of pneumonia of viral etiology, including SARS-CoV-2 infection, patients may present cardiac involvement, including myocarditis and arrhythmia [4]. As the severity of CAP may range from being almost asymptomatic to extremely severe, with septic shock and multiple system organ failure syndrome (MSOF), standardization of assessment of the disease severity, the likelihood of associated risks, and the prognosis are necessary. Various prognostic scoring systems have been developed, but these have failed to reach a consensus and are not universally recognized, with implications for the optimization of therapeutic management [5,6]. The ultimate goal is better monitoring of the clinical status and the prognosis of the patient in order to plan management in adapting the antibiotic regimen, with the aim of achieving a rapid response and reduction in the duration of hospitalization [7,8,9]. In order to increase the efficacy of prognostic scores in CAP, the inflammatory and immune biomarkers that most clinicians rely on should be taken into account. Currently, among the plethora of markers proposed for evaluating patients with severe infections, those that have been most comprehensively evaluated are the acute-phase proteins, represented by C-reactive protein (CRP) and procalcitonin (PCT) [10].

The innate immune response is the first line of defense of the body, acting within minutes via mobilization of the polymorphonuclear leucocytes, the complement system, and a specific group of cells with cytotoxic properties [11,12]. Mannose-binding lectin (MBL) is a polymorphic glycoprotein that belongs to the collectin family and is characterized by the presence of both collagen-like substances and lectin in the same subunit. Synthesized in the liver, MBL was the first protein discovered to play an important role in initiating the lectin pathway of complement activation [13]. This pathway is distinguished by the trigger mechanism, which is activated when microorganisms with multiple mannan-type polysaccharide residues enter the system [13]. MBL has been documented to interact with a wide variety of encapsulated Gram-positive and Gram-negative bacteria, viruses, yeasts, mycobacteria, parasites, and protozoa. MBL is considered to be a pre-antibody with a defensive role, particularly important in the early stages of life when the adaptive immune system is still immature. The serum levels are genetically predetermined, and low levels of MBL indicate an immunodeficiency defect, while raised levels indicate an active infection [13]. MBL deficiency has been reported to be associated with increased susceptibility to infectious diseases, especially those affecting the respiratory system [13], but the publications to date were based on limited data, and additional scientific evidence is needed.

The main objective of this study was a comparative analysis of the efficacy of CRP, PCT, and MBL in the assessment of the prognosis in children diagnosed with CAP.

## 2. Materials and Methods

### 2.1. Study Design

A retrospective, observational study was conducted on patients with CAP admitted to the “Maria Skłodowská Curie” Emergency Children’s Hospital in Bucharest between 2018 and 2020. This clinical unit is the largest emergency hospital for children in Romania, serving a population of approximately 2 million inhabitants in the immediate vicinity, but also patients with severe diseases from more distant areas. The study was performed in accordance with the strengthening of the reporting of observational studies in epidemiology (STROBE) guidelines [14].

The criteria for inclusion in the study comprised age between 4 months and 17 years, diagnosis of CAP, according to the criteria shown in Table 1 [15], availability of CRP and PCT measurements within the first 24 h of hospitalization, and MBL within the first 72 h of hospitalization (Table 1). The exclusion criteria included a diagnosis of asthma, autoimmune disease, chronic neurological disorder, and primary or secondary immunodeficiency, and discharge from hospitalization in a healthcare facility ≤ 14 days before the onset of symptoms of CAP.

**Figure 1:**
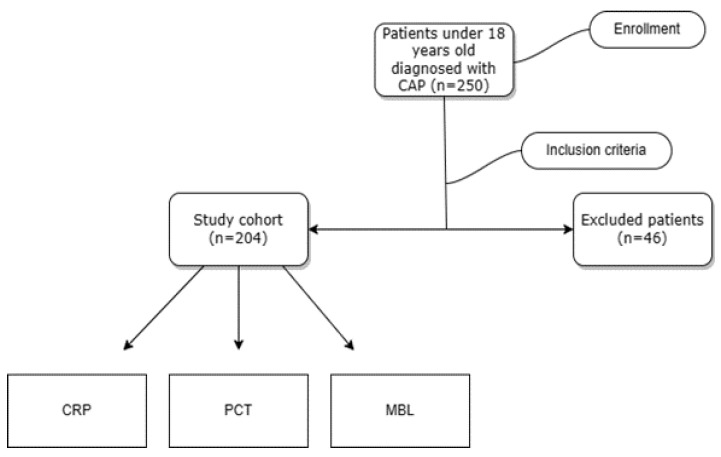
Flow chart of the study of children hospitalized with community-acquired pneumonia (CAP) between 2018 and 2020. MBL: mannose-binding lectin, CRP: C-reactive protein, PCT: procalcitonin.

Initially, the records of 250 patients hospitalized with CAP were retrieved, but, as shown in the flowchart (Figure 1), 46 did not meet the study criteria. In accordance with the inclusion criteria, a total of 204 patients aged 4 months to 17 years, with a median age of 42 months, with a positive diagnosis of CAP, were identified and included in the study cohort. The relevant data were retrieved from the hospital’s medical records. The decision for hospitalization was taken in the emergency department based on the personal medical history, clinical examination, and laboratory and imaging findings. After hospitalization, the patients underwent additional tests relevant to the disease, such as CBC (complete blood count) tests, CRP, and PCT. MBL values are available for all of the patients included in this retrospective study as they originate from a significantly larger cohort of children hospitalized in our university clinic, whose levels of MBL were analyzed within other prospective studies in order to assess the relationship between MBL deficit and prognosis of lower respiratory tract disease. All custodians of the participating patients signed a consent form at admission, granting approval of the collection of data that is relevant to the evaluation of the relationship between MBL and lower respiratory tract infections’ prognosis. Therefore, this study was conducted according to the ethical standards of observational studies, and approval was obtained from the Ethics Committee of the hospital.

### 2.2. Blood Sampling

Venous blood samples were collected from the older children using special vacutainer tubes containing the anticoagulant tripotassium/dipotassium/disodium ethylenediaminetetraacetic acid (EDTA). Infants underwent blood collection from finger capillaries using heparin-rich microtainers. The blood count was determined on an autoanalyzer based on flow cytometry. The blood samples were centrifuged to separate serum or plasma from the other blood components.

### 2.3. Biomarker Analysis

CRP was measured using a photometric turbidimetry immunoassay with a semiautomatic chemistry analyzer—COBAS 6000 (Roche Diagnostics, Meylan, France). PCT was measured using electrochemiluminescence immunoassay “ECLIA” (Elecsys^®^ BRAHMS) using COBAS E411. MBL was measured with the use of the solid phase “sandwich” variant of enzyme-linked immunosorbent assay (ELISA) by means of diagnostic kits Human MBP-C/MBL2 (mannose-binding protein C/mannose-binding lectin) ELISA Kit LS-F21071.

### 2.4. Biomarker Reference

The reference value of CRP, according to the laboratory method, is 5 mg/L [16]. For the purposes of this study, a cutoff value of 100 mg/L was used, as, according to the relevant literature and clinical practice, a level of CRP of above 100 mg/L is closely related to severe disease [17,18,19]. In acute bacterial infections, the level of CRP increases rapidly after four to six hours, reaching values above 100 mg/L, and decreases within a few days of successful treatment of the infection [20].

The laboratory detection test for PCT, the Elecsys^®^-BRAHMS method, relies on a sandwich immunoassay principle, utilizing two monoclonal antibodies specific to PCT. These antibodies bind to distinct epitopes on the PCT molecule, ensuring high specificity and sensitivity. The assay is automated and performed on Roche’s Elecsys^®^ immunoassay systems, streamlining the testing process and providing a quantitative expression of PCT, typically within 20 min, enabling timely clinical intervention [21]. For the purposes of this study, in order to stratify the risk of severe infection, the following PCT cutoff values were used [22,23]:

- <2 ng/mL: Systemic infection is possible, but unlikely;
- 2–10 ng/mL: Systemic infection is likely;
- ≥10 ng/mL: High likelihood of severe bacterial sepsis or septic shock.

To measure MBL, venous blood was collected within the first 72 h of hospitalization. The laboratory reference value for MBL is 450 ng/mL; thus, a level below this threshold indicates an immune deficiency [16].

### 2.5. Classification

The patient cohort was variously grouped into categories based on the levels of the biomarkers, as follows:

Group A comprised patients with a CRP level of <100 mg/L, and Group B comprised patients with a CRP level of ≥100 mg/L. 

Patients with a PCT level of <2 ng/mL were placed in Group X, those with PCT levels between 2 and 10 ng/mL in Group Y, and those with PCT levels ≥10 ng/mL in Group Z.

Group α included patients with a low level of MBL (<450 ng/mL), and Group β patients with MBL levels within the normal range.

The data recorded relevant to the prognosis and outcome of CAP include the following: peripheral oxygen saturation (SpO2%), progression to respiratory failure, development of complications, duration of antibiotic treatment, antibiotic combinations, need for oxygen therapy, surgical intervention, transfer to the intensive care unit (ICU), duration of hospitalization, and overall outcome. These are parameters that have been evaluated in other studies on the prognosis of CAP in children [22].

### 2.6. Statistical Analysis

Statistical analysis was performed using special data management software: Analyze-it (Microsoft Excel v16.0) and SPSS IBM v.26 (Chicago, IL, USA). To evaluate the normality assumption of the distribution of the variables, the Shapiro–Wilk test was performed on the dataset. Additionally, graphical methods were employed, including the examination of histograms overlaid with distribution curves, Q-Q plots, and box plots, to offer visual insight. Careful examination of the results from both the Shapiro–Wilk tests and the graphical representations showed that the data did not conform to a normal distribution. Summary statistics are presented as the number of observations, frequencies, median, minimum and maximum values, and the interquartile range (IQR) with 25th–75th percentile values (first and third quartiles). Comparison within groups was assessed using nonparametric tests—the chi-squared test of independence and Fisher’s exact test for categorical variables. The Wilcoxon–Mann–Whitney test and Kruskal–Wallis test were conducted to compare numerical variables. Sensitivity and specificity were reported for each biomarker, depending on the best performance as predictors for different events, using logistic regression. The discriminatory effectiveness was evaluated by analyzing receiver operating characteristic (ROC) curves and interpreted quantitatively using the area under the ROC curve (AUC) values. The optimal threshold for each parameter was established using Youden’s J statistic tests. Statistical significance was considered if the p-value was less than 0.05, 95% confidence interval (CI) in two-tailed tests.

## 3. Results

### 3.1. Baseline Characteristics

The study cohort consisted of 204 children, ranging in age between 4 months and 17 years, with a median age of 42 months, who had been hospitalized with a diagnosis of CAP. The demographic characteristics, symptoms, and baseline clinical findings of the study children are shown in Table 2, and the laboratory and imaging findings in Table 3.

### 3.2. Cohort Analysis Based on CRP Levels

**Figure 2:**
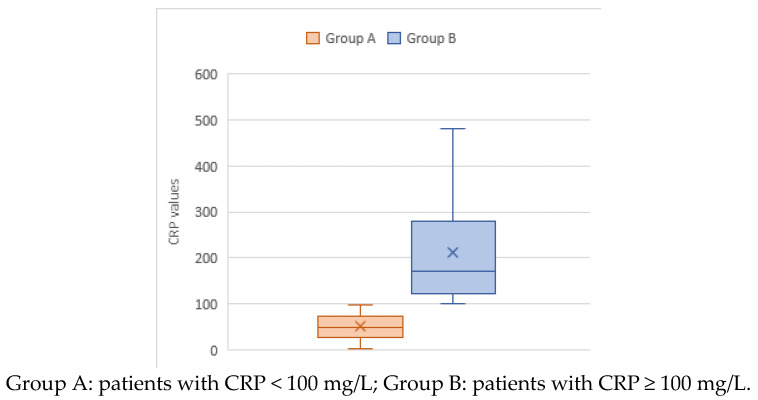
Box plot of the distribution of levels of C-reactive protein (CRP) in children hospitalized with community-acquired pneumonia (N = 204). The “×” sign indicates the mean value.

The distribution of the levels of CRP in the study cohort is presented in box-plot form in Figure 2, according to Groups A and B, with low (<100 mg/L) and high levels, respectively. The highest level of CRP in Group A was 98 mg/L, and the lowest was 1.5 mg/L, with a median of 50 mg/L and IQR 27.572 mg/L. In Group B, the minimum CRP level was 100 mg/L, the maximum was 481 mg/L, and the median was 170 mg/L (IQR = 121.7–278.7). The mean CRP level in Group A was 50.85 mg/L; in Group B, it was 212.6 mg/L.

As shown in Table 4, only 7.36% of the patients in Group A (CRP <100 mg/L) presented acute respiratory failure, compared with 32.11% in Group B (CRP ≥100 mg/L). In Group A, 17.9% developed local complications, such as pleurisy, necrotizing pneumonia, and lung abscess, compared with 36.7% in Group B (*p* < 0.05). In Group A, 10.5% developed systemic complications, namely, inflammatory response syndrome (SIRS), sepsis, or septic shock, compared with 78.9% in Group B (*p* < 0.05). Patients in Group A received antibiotic treatment for a median of 7 days, compared with 10 days for patients in Group B (*p* < 0.05). Approximately 6.31% of the patients in Group A and 32.11% of the patients in Group B required oxygen therapy. Surgical intervention was performed in 2.1% of the patients in Group A, compared with 12.84% in Group B (*p* < 0.05). In Group B, 9.17% of the patients were admitted to the intensive care unit (ICU) and required mechanical ventilation, compared with only 2.21% of the Group A patients (*p* < 0.05). The median duration of hospital stay was 7 days for patients in Group A and 10 days for patients in Group B.

**Figure 3:**
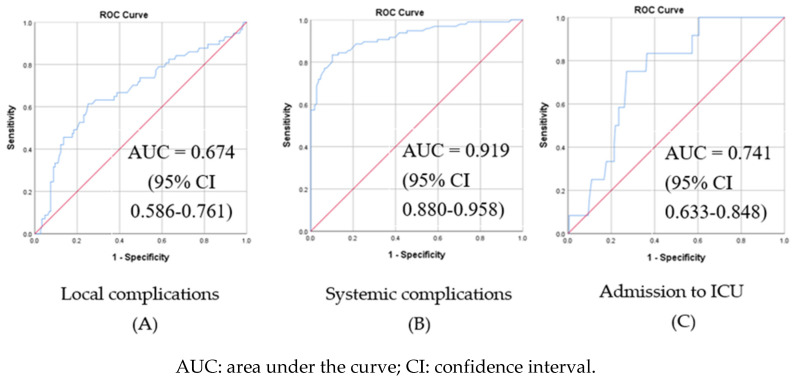
Receiver operating characteristic (ROC) curves for C-reactive protein (CRP) as a predictor of local (**A**) and systemic (**B**) complications of pneumonia and admission to ICU (**C**) in children hospitalized for community-acquired pneumonia (N = 204).

Figure 3 shows the ROC curves for CRP in the study population. CRP performed best as a predictor of systemic complications (SIRS, sepsis, and septic shock), with excellent discrimination (AUC= 0.919, 95% CI = 0.880–0.958), while for the other two conditions (local complications and admission to the ICU), the discrimination level appears to be poor (AUC = 0.674 and 0.741, respectively). This means that the CRP is a relatively weak predictor of local complications and admission to the ICU, although the *p*-value is <0.0001, which demonstrates statistical significance. The optimal threshold for CRP as a predictor of systemic complications was determined at 115 mg/L (Youden’s J index). According to this threshold, the sensitivity of this test is 83%, and the specificity is 90%.

### 3.3. Cohort Analysis Based on PCT Levels

In the study cohort, the maximum recorded level of PCT was 40 ng/mL, and the minimum was 0.01 ng/mL with a mean of 4.96 ng/mL (within Group Y) and a median of 0.485 ng/mL (IQR = 5.415).

**Figure 4:**
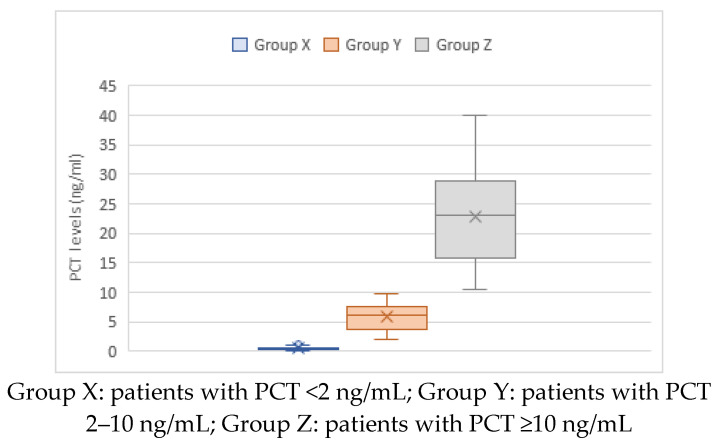
Box plot of the distribution of levels of procalcitonin (PCT) in children hospitalized with community-acquired pneumonia (N = 204). The “×” sign indicates the mean value.

Figure 4 shows box plots of the distribution of PCT in three groups: Group X ranged from 0.01 ng/mL to 1.99 ng/mL, with a median of 0.3 ng/mL (IQR = 0.2–0.537) and a mean of 0.47 ng/mL. PCT in Group Y ranged from 2.1 ng/mL to 9.8 ng/mL, with a median of 6.2 ng/mL (IQR = 3.8–7.5) and a mean of 5.8 ng/mL. Group Z had a minimum PCT of 10.5 ng/mL, a maximum of 40 ng/mL, a median of 23 ng/mL (IQR = 15.92–29 ng/mL), and a mean of 22.39 ng/mL.

Table 5 shows the course of the patients according to their levels of PCT. The patients in Group X, with the lowest levels, had an incidence of acute respiratory failure of 10.63%, compared with 20.69% in Group Y and 61.76% in Group Z (those with the highest levels). Local complications were present in 50% of the Group Z patients, with a lower incidence in Groups X and Y (19.58% and 41.37%, respectively). All the patients in Group Z, and the majority in Group Y, developed systemic complications (SIRS, sepsis, or septic shock), significantly higher than those in Group X. Patients in Group X required a median duration of 7 days of antibiotic treatment (minimum 3 days and maximum 30 days), compared with Group Y, with a median duration of 10 days (minimum 5 days, maximum 26 days), and Group Z, with a median of 14 days (minimum 7 days, maximum 35 days) (*p* < 0.05). In Group X, 9.92% of the patients required oxygen therapy, compared with 20.69% in Group Y and 61.76% in Group Z. Surgical intervention was performed in 2.12% of the children from Group X, 10.34% from Group Y, and 29.41% from Group Z (*p* < 0.05). Among the patients in Group Z, 26.47% required ICU admission and mechanical ventilation, compared with 0.71% in Group X and 3.44% in Group Y (*p* < 0.05).

**Figure 5:**
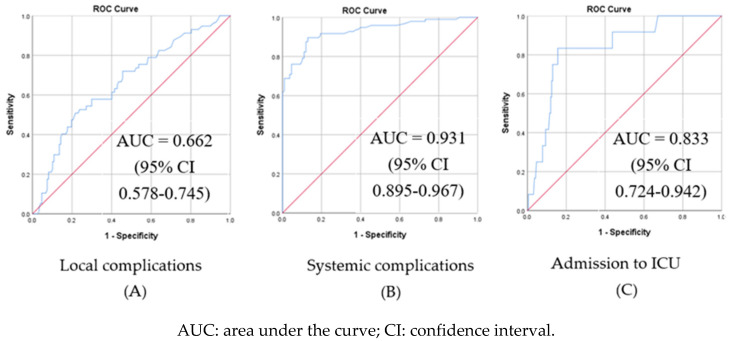
Receiver operating characteristic (ROC) curves for procalcitonin (PCT) as a predictor of local (**A**) and systemic (**B**) complications of pneumonia and admission to ICU (**C**) in children hospitalized for community-acquired pneumonia (N = 204).

The ROC for PCT is shown in Figure 5. PCT performed best as a predictor of systemic complications (SIRS, sepsis, and septic shock), with very significant discrimination power (AUC= 0.931, 95% CI = 0.895–0.967). In this cohort, PCT was a poor predictor of local complications (AUC = 0.662, 95%CI = 0.578–0.745). The optimal threshold determined through Youden’s J index for PCT as a predictor of systemic complications showed that a PCT threshold value of 0.5 ng/mL renders a sensitivity of 90% and specificity of 87%.

### 3.4. Cohort Analysis Based on MBL Levels

**Figure 6:**
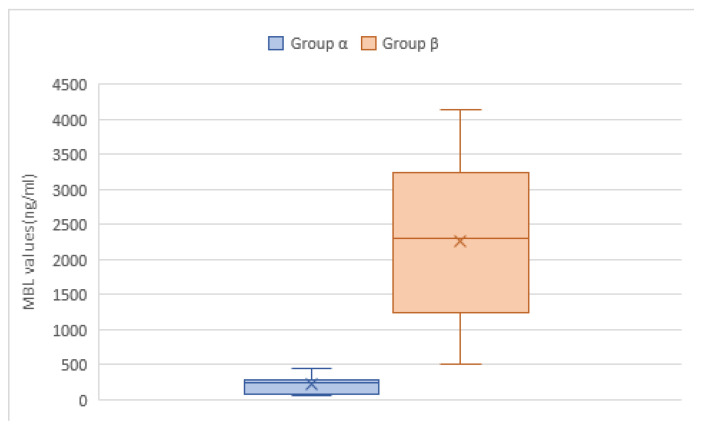
Box plot of the distribution of levels of mannose-binding lectin (MBL) in children hospitalized with community-acquired pneumonia (N = 204). The “×” sign indicates the mean value.

Figure 6 shows the box plot for the distribution of the levels of MBL in the study population, which ranged from 50 ng/mL to 4150 ng/mL, with a mean of 1877.6 ng/mL and a median value of 1580 ng/mL (IQR = 4100 ng/mL). In Group α, the minimum MBL level was 50 ng/mL, the maximum 449 ng/mL, the median 250 ng/mL (IQR = 76–292 ng/mL), and the mean 220 ng/mL. In Group β (the reference group, MBL ≥ 450 ng/mL), the minimum value of MBL is 508 ng/mL, while the maximum one was 4150 ng/mL. The median level of MBL was 2300 ng/mL (IQR = 1245.8–3250 ng/mL), with a mean of 2256.6 ng/mL.

The included 37 patients had MBL levels below the threshold of 450 ng/mL, of which 24.32% had a level of <50 ng/mL, indicating complete deficiency. Table 6 shows the association of the clinical features and course of CAP with the levels of MBL in the study children, low (Group α) or high (Group β).

In Group α, 56.75% of the patients were diagnosed with acute respiratory failure, compared with 11.97% in Group β (*p* < 0.05). Group α patients with MBL deficiency had a higher incidence of local complications (48.64%) and systemic complications (83.78%) than those in Group β (23.35% and 38.92%, respectively) (*p* < 0.05). The median duration of antibiotic treatment was twice as long in patients with MBL deficiency (14 days vs. 7 days) (*p* < 0.05). In Group α, 51.35% of the patients required supplemental oxygen, with a median duration of 3 days, compared with 13.17% in Group β, with an average duration of 2 days. In Group α, 24.32% of the patients underwent surgery, compared with only 4.19% in Group β (*p* < 0.05) and 21.62% of the Group α patients, but only 2.39% of the Group β patients were transferred to the ICU and required mechanical ventilation. Regarding the duration of hospital stay, the median number of days was 14 for Group α patients, whereas it was 7 for Group β patients. 

**Figure 7:**
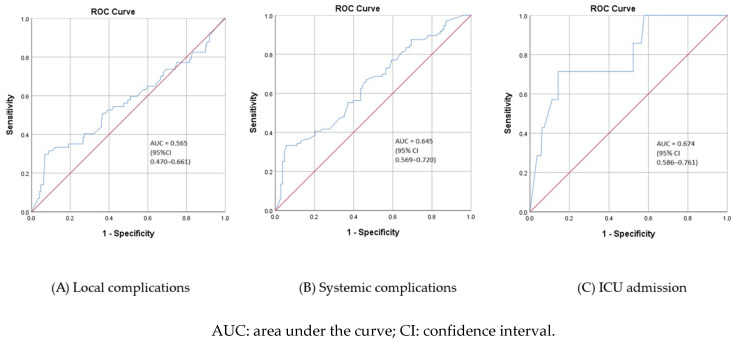
Receiver operating characteristic (ROC) curves for mannose-binding lectin (MBL) as a predictor of local (**A**) and systemic (**B**) complications of pneumonia and admission to ICU (**C**) in children hospitalized for community-acquired pneumonia (N = 204).

Figure 7 shows the ROC curves for MBL according to the incidence of local complications, systemic complications, and admission to the ICU of children hospitalized with CAP. MBL proved to be a relatively poor predictor of local complications, systemic complications, and admission to the ICU, as all the estimated AUC values are below 0.8. However, MBL tends to perform the best as a marker of CAP that needs treatment within the ICU (AUC = 0.797, 95% CI 0.629–0.965). The optimal threshold for MBL, according to Youden’s J statistic, was 50, with corresponding sensitivity and specificity of 96% and 25%, respectively. 

## 4. Discussion

CAP is the leading infectious cause of death in children under the age of five years worldwide [23]. One method by which clinicians can assess the severity of a respiratory infection is by using inflammatory markers. The findings on clinical examination, together with the CRP level, are useful in the diagnosis of patients with severe bacterial infections, such as sepsis, pneumonia, meningitis, osteomyelitis, cellulitis [24,25], peritonitis [26], and bone and joint infections [27]. In the case of lower respiratory tract infections, CRP has been studied and utilized for its ability to differentiate between bacterial and viral pathogens [28].

In this retrospective study, we demonstrated that a raised level of CRP (>100 mg/L) in children diagnosed with CAP is associated with a higher incidence of both local complications (pleural effusion, necrotizing pneumonia, and lung abscess) and systemic complications (SIRS, sepsis, and septic shock). The ROC curves of CRP and presentation of local and systemic complications and ICU admission confirmed a strong relationship (*p* < 0.05), with CRP performing best as an indicator of systemic complications of CAP (AUC = 0.919).

In addition, the children with raised CRP required a longer duration of antibiotic therapy and more often required surgical intervention, transfer to the ICU, intubation, and mechanical ventilation. The duration of hospitalization was longer for children with CRP ≥ 100 mg/L (median 10 days) than for those with lower levels of CRP (median 7 days).

These findings are similar to those documented in other studies [29,30,31], adding to the body of evidence supporting the use of inflammatory markers in the management of patients diagnosed with CAP, as they have a predictive role in disease progression. A raised CRP is more frequently associated with the development of pleural effusion, bacteremia, ICU admission, mechanical ventilation, septic shock, and death [26]. Our study emphasizes, once again, the usefulness of CRP in the evaluation and prognosis of CAP in children, demonstrating that a CRP level of above 100 mg/L is associated with an increased risk of complications.

Despite studies that demonstrate limitations in evaluating severe infections using biomarkers, our results regarding sensitivity and specificity clearly favor PCT in comparison to CRP [32] in terms of prediction of systemic complications: AUC = 0.919 for CRP vs. AUC = 0.931 for PCT, together with 90% specificity and 83% sensitivity for CRP, and 90% sensitivity and 87% specificity for PCT (Table 7).

Numerous studies have been conducted to evaluate the impact of various different markers involved in the inflammatory process and their relationship with mortality. The results have confirmed that high levels of PCT are closely related to bacterial infection and its complications and are a predictive factor for a fatal outcome [33]. A particularly strong relationship between PCT levels and the presentation of systemic complications has also been documented [34,35].

It is apparent that early measurement of PCT can enable stratification into risk groups and identify potentially life-threatening infections. As a predictive factor for infection severity, PCT can provide guidance for the duration of antibiotic treatment and the need for antibiotic combinations [33,34,35,36,37,38,39,40]. In our study, PCT levels of above 10 ng/mL provided rapid information about the progression and prognosis of patients diagnosed with CAP: raised levels were clearly associated with disease severity and the presentation of systemic complications, including SIRS, sepsis, and septic shock. PCT measurement as an index of the severity of CAP bacterial infection at all ages can assist the physician in choosing appropriate management from the time of hospital admission.

In this study, the role of MBL deficiency in the presentation of complications of CAP was observed, which is in line with research conducted on laboratory animals regarding the role of MBL in susceptibility to pneumococcal infection [41,42]. A clinical study confirmed this relationship, reporting a significant association between MBL deficiency and increased risk of acute respiratory infection in children aged 6 to 17 months [43]. Data from another study contradict this, as no correlation was observed in vitro between MBL deficiency and/or MBL-mediated opsonophagocytosis for *Streptococcus pneumoniae* [44]. The classical pathway of complement is the main pathway involved in defense against pneumococcus, and MBL deficiency does not appear to significantly affect the immune response of laboratory animals to this type of infection [45]. Other explanations for the ways in which MBL deficiency may influence the progression and prognosis of bacterial CAP are based on its role in apoptosis or its ability to inhibit the production of proinflammatory cytokines induced by macrophages [45].

Although the classical pathway of complement activation was initially proposed as the main biological mechanism responsible for the opsonization of bacteria, it did not compensate for the deficiency in the lectin pathway [46]. The same pattern was observed for the alternative pathway of complement activation. Laboratory animals with MBL deficiency were observed to be highly vulnerable to bacterial infection, and the opsonization process failed, resulting in them exhibiting survival periods and mortality rates similar to those observed in deficiency of the C1q and C4 fractions [46].

In our study, the group with MBL deficiency included more patients diagnosed with sepsis and septic shock compared with the group without MBL deficiency. Initial research on the role of MBL in sepsis indicated that this molecule can bind to lipopolysaccharides, leading to the activation of the C4 fraction and causing the development of septic shock in laboratory animals [13].

In a study on 114 patients diagnosed with septic shock and 81 with sepsis, those with MBL deficiency recorded higher sequential organ failure assessment (SOFA) scores, while those with high levels of MBL levels showed favorable SOFA results [44]. Another study on patients with septic shock reported that those with an increase of over 10% in the level of MBL concentration within 6 h of the diagnosis of septic shock had a significantly lower mortality rate than those whose MBL level decreased by over 10% during the same period. MBL deficiency and its decrease in the early phase of septic shock may, therefore, be associated with increased mortality [47].

The lack of consistency in the literature regarding the impact of MBL deficiency in patients with severe infections can have various explanations. Firstly, the population in each report originates from different geographical areas, and environmental factors and genetic background diversity could influence the frequency of polymorphisms. Secondly, the small sample size in some studies contributes to the generation of divergent results, and larger samples will be needed to reduce the possibility of false-positive or false-negative associations. Thirdly, heterogeneity in the composition of control populations in the various studies can influence the susceptibility to sepsis.

In our study, the children diagnosed with CAP who had MBL deficiency developed sepsis and septic shock more frequently than those without MBL deficiency. The children with MBL deficiency, in addition to a higher frequency of complications and need for surgical intervention, also had a higher likelihood of ICU admission and mechanical ventilation requirement and a slower resolution of infection.

At the onset of severe infections, inflammatory markers can guide therapeutic decisions, and based on their levels, a complex treatment regimen can be initiated. It is recognized that complicated pneumonia may require treatment for more than 14 days. Measurement of MBL at a later stage, specifically at 7 days after the initial collection, can indicate the possible need for more aggressive and prolonged antibiotic treatment.

At the onset of an infection, the level of MBL increases, playing a protective role against the action of the pathogen [48]. It has been demonstrated that MBL and CRP can be used as paraclinical biomarkers to predict the outcome of CAP [48]. A total of 104 patients diagnosed with CAP and 100 healthy individuals were evaluated. The levels of MBL and CRP were recorded in the patients before the initiation of antibiotic treatment and on days four and seven of treatment. Compared with the control group, the levels of MBL and CRP were significantly higher in patients diagnosed with CAP, with the CRP level decreasing within one week and the MBL level persisting beyond this period [48]. A study of neonatal sepsis confirmed the association between raised levels of MBL and specific markers of severe infections, including CRP, PCT, and interleukin-6 (IL-6) [49].

Measurement of the MBL level at the onset of an infection is particularly recommended in children at high risk. For patients with significant comorbidities, such as asthma [50], congenital heart malformations [51], and chronic kidney disease [52], a timely and appropriate therapeutic approach is vital, as any acute infective episode can lead to potentially life-threatening decompensation. The blood level of MBL at the onset of an infection can identify deficiency, indicating higher risk, and thus ensure the selection of optimal treatment for this vulnerable patient group.

## 5. Limitations of This Study

The levels of biomarkers should always be interpreted in a clinical and microbiological context. Since both diagnosis and prognosis are important, the dynamics of biomarkers should be assessed through serial measurements, especially in patients with CAP who present local or systemic complications. Our retrospective study, however, was not designed to track the progression of the illness in terms of biomarker dynamics.

Another important aspect is that the results of the inflammatory markers, CRP and PCT, are available within a few hours, allowing for therapeutic decisions to be influenced and, consequently, the treatment would have affected the progression and prognosis of the disease.

Regarding the level of MBL, which could indicate a possible immune deficiency and the need for more aggressive therapy from the onset of the disease, this was available only after 7 days of collection. This timing only influenced the therapeutic approach in cases with prolonged evolution, although the levels were of great research interest.

It should be noted that the results are based on the data obtained retrospectively from the specific study cohort and are to be interpreted considering the limitations and methodology of the study.

## 6. Conclusions

This retrospective study, conducted on 204 children aged between 6 months and 17 years who were hospitalized with CAP, revealed the significance of the levels of CRP, PCT, and MBL in assessing the potential risk of local and systemic complications and the need for surgical intervention, ICU admission, and prolonged hospitalization.

These findings provide additional evidence regarding the efficacy of these three markers in determining an appropriate therapeutic approach for children with CAP. We believe that early assessment of these markers has the potential to improve the management and, thus, the prognosis and outcome of these patients.

## Figures and Tables

**Table 1:** Diagnosis of community-acquired pneumonia (CAP) in immunocompetent children.

CAP Is an Acute, Symptomatic Infection of the Pulmonary Parenchyma in a Child Who Has Not Been Hospitalized in a Healthcare Facility for ≤14 Days before the Onset of Symptoms. The Diagnosis Requires Two Clinical Findings Plus Fever and Tachypnoea, with Laboratory and Radiographic Confirmation.
Clinical findings (≥two of the following)
-Cough
-New onset of lower respiratory tract secretions, change in character of secretions, or increase in the quantity or suctioning requirements
-Auscultatory findings of consolidation (rales, bronchial breath sounds, egophony, decreased breath sounds)
-Dyspnoea
Vital signs (mandatory)
-Fever defined by age group
-Tachypnoea defined by age group
Laboratory findings
-White blood cell count (WBC) ≥15,000 or ≤4000 per cubic millimeter
Radiographic findings
-Chest X-ray revealing the presence of new infiltrate(s) consistent with infection (interstitial, bronchial, alveolar), consolidation, cavitation, abscess, or pneumatoceles

**Table 2:** The demographic characteristics, medical history, and clinical findings of children hospitalized with community-acquired pneumonia (N = 204).

Parameters	Distribution
Male (%)	101 (49.5%)
Age (months)—median [min, max]	42 [4, 212]
Onset of fever (days)—median [min, max]	3 [1, 14]
Onset of cough (days)—median [min, max]	5 [1, 21]
Vaccination status—*n* (%)	188 (92.15%)
Fever (degrees Celsius)—median [min, max]	39 [37, 41]
Altered mental status—*n* (%)	5 (2.45%)
SpO2%—median [min, max]	96% [60%, 99%]
Respiratory rate (breaths per minute)—median [min, max]	30 [20, 75]
Apnea—*n* (%)	3 (1.47%)
Grunting—*n* (%)	23 (11.27%)
Thoracic pain—*n* (%)	17 (8.33%)
Cyanosis—*n* (%)	8 (3.92%)
Tachycardia—(%)	77 (37.74%)
Capillary refill ≥ 3 s—*n* (%)	3 (1.47%)
Respiratory failure—*n* (%)	42 (20.58%)
Local complications—*n* (%)	57 (27.94%)
• Pleurisy—*n* (%)	49 (24.01%)
• Abscess—*n* (%)	7 (3.43%)
• Necrotizing pneumonia—*n* (%)	1 (0.49%)
Systemic complications—*n* (%)	96 (47.05%)
• SIRS—*n* (%)	35 (17.15%)
• Sepsis—*n* (%)	49 (24.01%)
• Septic shock—*n* (%)	12 (5.88%)
Antibiotic treatment (days)—median [min, max]	7 [3, 35]
Oxygen therapy—*n* (%)	41 (20.09%)
Oxygen therapy (days required)—median [min, max]	2 [1, 7]
Surgical intervention (%)	16 (7.84%)
• Chest drainage—*n* (%)	7 (3.43%)
• Lung decortication—*n* (%)	4 (1.96%)
• Lobectomy—*n* (%)	5 (2.45%)
Admission to the ICU—*n* (%)	12 (5.88%)
Duration of ICU stay (days)—median [min, max]	1 [1, 14]
Death—*n* (%)	3 (1.47%)
Duration of hospitalization (days)—median [min, max]	7 [3, 35]

SIRS: systemic inflammatory response syndrome, ICU: intensive care unit. Note: Consolidation—accumulation of inflammatory cellular exudate in the alveoli and adjoining ducts; pleurisy—inflammation of the pleura, usually occurring as a complication of a disease such as pneumonia.

**Table 3:** Laboratory and imaging findings of children hospitalized with community-acquired pneumonia (N = 204).

Paraclinical Parameters	Distribution
MBL (ng/mL)—median [min, max]	1580 [50, 4150]
• Low (<450 ng/mL)—*n* (%)	37 (18, 13%)
• Normal (≥450 ng/mL)—*n* (%)	167 (81, 86%)
White blood cells (cells/µL)—median [min, max]	17,615 [1300, 57,980]
• Neutrophils—median [min, max]	77% [15, 92]
• Lymphocytes—median [min, max]	14% [2, 55]
Platelets (cells/µL)—median [min, max]	334,000 [10,000, 1, 100,000]
CRP (mg/L)—median [min, max]	100.5 [1.5, 481]
• CRP < 100 mg/L—*n* (%)	95 (46.56%)
• CRP ≥ 100 mg/L—*n* (%)	109 (53.41%)
PCT (ng/mL)—median [min, msax]	0.485 [0.01, 40]
• PCT < 2 ng/mL—*n* (%)	141 (69.11%)
• 2 ≤ PCT < 10 ng/mL—*n* (%)	29 (14.21%)
• PCT ≥ 10 ng/mL—*n* (%)	34 (16.6%)
Creatinine (mg/dl)—median [min, max]	0.3 [0.1, 5.2]
Urea (mmol/L)—median [min, max]	2.34 [0.83, 9.96]
Elevated liver enzymes (%) *n* (%)	9 (4.41%)
APTT (seconds)—median [min, max]	24 [12, 64]
Imaging findings (X-ray/Thoracic ultrasound/CT)—*n* (%)	
• Single lobe consolidation—*n* (%)	146 (71.56%)
• Multilobar consolidation—*n* (%)	18 (8.82%)
• Abscess—*n* (%)	7 (3.43%)
• Pleurisy—*n* (%)	49 (24.01%)

MBL: mannose-binding lectin, CRP: C-reactive protein, PCT: procalcitonin, APTT: activated partial thromboplastin time, CT: computed tomography. Note: Consolidation—accumulation of inflammatory cellular exudate in the alveoli and adjoining ducts; pleurisy—inflammation of the pleura, usually occurring as a complication of a disease such as pneumonia.

**Table 4:** Clinical course of children hospitalized with community-acquired pneumonia (N = 204), according to the level of C-reactive protein (CRP).

Clinical Feature	Group A CRP < 100 mg/L; N = 95	Group B CRP ≥ 100 mg/L; N = 109	*p* Value *
Peripheral oxygen saturation %—median [min, max]	97% [65%, 99%]	95% [60%, 99%]	0.00128
Acute respiratory failure—*n* (%)	7 (7.36%)	35 (32.11%)	0.00076
Incidence of local complications—*n* (%)	17 (17.9%)	40 (36.7%)	0.002832
Pleurisy—*n* (%)	16 (16.84%)	33 (30.27%)	0.025072
Lung abscess—*n* (%)	1 (1.05%)	6 (5.5%)	0.081431
Necrotizing pneumonia—*n* (%)	0 (0%)	1 (0.9%)	1
Incidence of systemic complications—*n* (%)	10 (10.5%)	86 (78.9%)	0.00001
SIRS—*n* (%)	6 (6.31%)	29 (26.6%)	0.000126
Sepsis—*n* (%)	3 (3.15%)	46 (42.2%)	0.00001
Septic shock—*n* (%)	1 (1.05%)	11 (10.09%)	0.0062
Duration of antibiotic therapy (days)—median [min, max]	7 [3, 25]	10 [4, 35]	0.00001
Combination of antibiotics—median [min, max]	1 [1, 3]	2 [1, 4]	0.00001
Oxygen therapy—*n* (%)	6 (6.31%)	35 (32.11%)	0.00001
Duration of oxygen therapy (days)—median [min, max]	0 [0, 3]	0 [0, 7]	0.00128
Surgical intervention—*n* (%)	2 (2.1%)	14 (12.84%)	0.00443
Admission to the ICU—*n* (%)	2 (2.1%)	10 (9.17%)	0.032317
Duration of ICU stay (days)—median [min, max]	0 [0, 10]	0 [0, 14]	0.00001
Death—*n* (%)	0 (0%)	3 (2.75%)	0.2499
Duration of hospitalization (days)—median [min, max]	7 [3, 25]	10 [4, 35]	0.00001

SIRS: systemic inflammatory response syndrome; ICU: intensive care unit. * Mann–Whitney U test was used for quantitative variables, while Chi-squared and Fisher’s test were used for qualitative variables.

**Table 5:** Clinical course of children hospitalized with community-acquired pneumonia (N = 204), according to the level of procalcitonin (PCT).

Parameter	Group X PCT < 2 ng/mL N = 141	Group Y 2 ng/mL < PCT< 10 ng/mL N = 29	Group Z PCT ≥ 10 ng/mL N = 34	*p* Value *
Peripheral oxygen saturation %—median [min, max]	97 [60, 99]	95 [77, 99]	91 [65, 98]	0.00001
Acute respiratory failure—*n* (%)	15 (10.63%)	6 (20.69%)	21 (61.76%)	0.00001
Incidence of local complications—*n* (%)	28 (19.58%)	12 (41.37%)	17 (50%)	0.000455
Pleurisy—*n* (%)	26 (18.43%)	11 (37.93%)	12 (35.29%)	0.026516
Abscess—*n* (%)	1 (0.7%)	1 (3.44%)	5 (14.7%)	0.00095
Necrotizing pneumonia—*n* (%)	1 (0.7%)	0 (0%)	0 (0%)	1
Incidence of systemic complications—*n* (%)	34 (24.11%)	28 (96.55%)	34 (100%)	0.00001
SIRS—*n* (%)	21 (14.89%)	12 (41.37%)	2 (5.88%)	0.000426
Sepsis—*n* (%)	13 (9.21%)	16 (55.17%)	20 (58.82%)	0.00001
Septic shock—*n* (%)	0% (0%)	0% (0%)	12 (35.29%)	0.00001
Duration of antibiotic treatment (days)—median [min, max]	7 [3, 30]	10 [5, 26]	14 [7, 35]	0.00001
Combination of antibiotics	1 [1, 4]	2 [1, 4]	2 [2, 4]	0.00001
Oxygen therapy—*n* (%)	14 (9.92%)	6 (20.69%)	21 (61.76%)	0.00001
Duration of oxygen therapy (days)—median [min, max]	0 [0, 7]	0 [0, 4]	1 [0, 5]	0.00001
Surgical intervention—*n* (%)	3 (2.12%)	3 (10.34%)	10 (29.41%)	0.00001
Admission to the ICU—*n* (%)	1 (0.7%)	1 (3.44%)	10 (29.41%)	0.00001
Duration of ICU stay (days)—median [min, max]	0 [0, 1]	0 [0, 2]	0 [0, 14]	0.07056
Death—*n* (%)	1 (0.7%)	0 (0%)	2 (5.88%)	0.13
Duration of hospitalization (days)—median [min, max]	7 [3, 30]	10 [0, 26]	14 [7, 35]	0.00001

* Kruskal–Wallis H test was used for quantitative variables, while Fisher’s test was used for qualitative variables.

**Table 6:** Clinical course of children hospitalized with community-acquired pneumonia (N = 204), according to the level of mannose-binding lectin (MBL).

Parameter	Group α MBL < 450 ng/mL; N = 37	Group β MBL ≥ 450 ng/mL; N = 167	*p* Value *
Peripheral oxygen saturation %—median [min, max]	92 [60, 98]	97 [72, 99]	0.00008
Acute respiratory failure—*n* (%)	21 (56.75%)	20 (11.97%)	0.00001
Incidence of local complications—*n* (%)	18 (48.64%)	39 (23.35%)	0.02
Pleurisy—*n* (%)	13 (35.13%)	36 (21.55%)	0.080246
Abscess—*n* (%)	5 (13.51%)	2 (1.19%)	0.000196
Necrotizing pneumonia—*n* (%)	0 (0%)	1 (0.59%)	1
Incidence of systemic complications—*n* (%)	31 (83.78%)	65 (38.92%)	0.00001
SIRS—*n* (%)	5 (13.51%)	30 (17.96%)	0.515888
Sepsis—*n* (%)	23 (62.16%)	34 (20.35%)	0.00001
Septic shock—*n* (%)	3 (8.1%)	1 (0.59%)	0.002875
Duration of antibiotic therapy (days)—median [min, max]	14 [7, 35]	7 [3, 10]	0.00001
Combination of antibiotics—median [min, max]	2 [1, 4]	1 [1, 4]	0.00001
Oxygen therapy—*n* (%)	19 (51.35%)	22 (13.17%)	0.00001
Duration of oxygen therapy (days)—median [min, max]	3 [1, 7]	2 [1, 5]	0.00001
Surgical intervention—*n* (%)	9 (24.32%)	7 (4.19%)	0.000038
Admission to the ICU—*n* (%)	8 (21.62%)	4 (2.39%)	0.00001
Duration of ICU stay (days)—median [min, max]	14 [7, 35]	0 [0, 5]	0.00001
Death—*n* (%)	2 (5.4%)	1 (0.59%)	0.028
Duration of hospitalization (days)—median [min, max]	14 [7, 35]	7 [3, 30]	0.00001

* Mann–Whitney U test was used for quantitative variables, while Chi-squared and Fisher’s test were used for qualitative variables.

**Table 7:** Cutoff values, sensitivity, and specificity reported for each biomarker in terms of local complications, systemic complications, and ICU admission.

	Local Complications			Systemic Complications			ICU Admission		
	CRP (mg/L)	PCT (ng/mL)	MBL (ng/mL)	CRP (mg/L)	PCT (ng/mL)	MBL (ng/mL)	CRP (mg/L)	PCT (ng/mL)	MBL (ng/mL)
Cutoff value	139	2.7	272	116	0.5	479	154	7.7	388
Sensitivity (%)	61%	51%	30%	83%	90%	33%	75%	75%	71%
Specificity (%)	74%	78%	32%	90%	87%	95%	73%	83%	86%

## Data Availability

Data is contained within the article.
